# Bioactive and degradable collagen-based three-dimensional scaffold encapsulated with adipose mesenchymal stem cells-derived exosomes improved diabetic wound healing

**DOI:** 10.1016/j.reth.2025.02.002

**Published:** 2025-02-20

**Authors:** Fereshteh Talebpour Amiri, Melody Omraninava, Shadman Shahzamani, Amirali Khodashenas, Arman Daryakar, Davood Nasiry

**Affiliations:** a Cellular and Molecular Research Center, Mazandaran University of Medical Sciences, Sari, Iran; b Hospital Administration Research Center, Sari Branch, Islamic Azad University of Medical Science, Sari, Iran; c Department of Clinical Science, Faculty of Specialized Veterinary Sciences, Science and Research Branch, Islamic Azad University, Tehran, Iran; d Student Research Committee, Mazandaran University of Medical Sciences, Sari, Iran; e Department of Medical Biotechnology, Faculty of Advanced Technologies in Medicine, Mazandaran University of Medical Sciences, Sari, Iran; f Department of Paramedicine, Amol School of Paramedical Sciences, Mazandaran University of Medical Sciences, Sari, Iran

**Keywords:** Diabetic wound, Wound healing, Collagen-based scaffold, Adipose mesenchymal stem cell, Exosomes

## Abstract

A diabetic wound is the most frequent form of chronic wound. Because diabetic wounds have multiple factors contributing to their development, the best treatments involve using a combination of approaches. Herein we assessed whether bioactive and degradable bioengineered micro-porous collagen-based three-dimensional scaffold (CTS) encapsulated with adipose mesenchymal stem cells (ASCs)-derived exosomes could accelerate the wound healing process in diabetic rats. Diabetic animals were assigned to the control group, CTS group, Exo group, and CTS+Exo group. Tissue samples were collected on days 7, 14, and 21 for evaluations including stereological, molecular, and tensiometrical assessments. The findings showed that in the treatment groups, there was a notably increase in wound closure rate, total volumes of newly formed epidermis and dermis, numerical densities of fibroblasts and blood vessels, collagen density, and biomechanical parameters than the control group, with the most noticeable changes seen in the CTS+Exo group. Additionally, there was a notably increase in the transcript of miRNA-146a, TGF-β, bFGF, and VEGF genes in the treatment groups than the control group, with the highest expression observed in the CTS+Exo group. In the CTS+Exo group, there was a much greater decrease in TNF-α and IL-1β expression, as well as in the number of neutrophils, compared to the other groups. These results validated that the combination of CTS and ASCs-derived exosomes has a greater effect on improving diabetic wound healing.

## Highlights

• The CTS encapsulated with ASCs-derived exosomes promote wound healing.
• The CTS encapsulated with ASCs-derived exosomes regulate the inflammatory process.
• The CTS encapsulated with ASCs-derived exosomes improve the stereological characteristics.
• The CTS encapsulated with ASCs-derived exosomes upregulated regenerative genes.
• The CTS encapsulated with ASCs-derived exosomes improve biomechanical characteristics.

## 1 Introduction

Diabetes mellitus is a prevalent and expensive chronic condition worldwide [1]. By 2030, it is estimated that the global population of individuals with diabetes mellitus will reach 700 million [2]. Following the disease's beginning, over time, individuals face various complications, with the most significant being the development of chronic wounds on their limbs [3]. In these individuals, healing wounds can be difficult as a result of high blood sugar levels, leading to oxidative stress, neuropathy, vasculopathy, and ongoing inflammation [4]. Hence, due to the various factors influencing chronic wounds, treatment should also involve multiple approaches.

Studies have indicated that the initial and crucial stage for healing diabetic wounds is to offer a proper porous scaffold for cells to penetrate and promote angiogenesis [5,6]. Until now, numerous scaffolds have been utilized for wound repair, however, biological scaffolds derived from the extracellular matrix (ECM) of native tissues have shown improved outcomes because of their resemblance to the dermis structure and safety [7]. The structure and makeup of ECM is an intricate blend of various proteins like glycoproteins, glycosaminoglycans, fibronectin, laminin, hyaluronan, and important cytokines for wound healing [8]. These factors play a crucial role in regulating various cellular functions. Recently, we explored if implanting dermal derived matrix could enhance wound healing in diabetic rats. We noticed that transplanting the dermal derived matrix-scaffold has a greater potential for improving the healing of diabetic wounds [9,10]. Consequently, we determined that the micro-porous bioengineered collagen-based three-dimensional scaffold (CTS) offers a more favorable microenvironment for cell penetration, aiding in wound healing.

Nevertheless, because diabetic wounds are complex, utilizing additional complementary compounds with the appropriate scaffold can result in synergistic and beneficial healing effects [6].

In recent years, a number of researches have highlighted the significant promise of adipose mesenchymal stem cells (ASC) for diabetic wound treatment [9,[11], [12], [13]]. However, it is thought that in these studies, ASCs' capacity to heal wounds is mainly attributed to the release of paracrine exosomes from these cells, rather than their versatile ability to differentiate.

Exosomes are extracellular vesicles that harbor many nucleic acids, proteins, and lipids. The contents vary with each cell type and physiologic status [14]. Recently, many studies indicated that ASCs-derived exosomes can be phagocytosed by macrophages in diabetic wounds to relieve oxidative stress and promote macrophage phenotype shift that inhibits excessive inflammation, promote angiogenesis, hasten local cell proliferation, and enhance collagen production, ultimately aiding in the healing of wounds [[15], [16], [17]].

Herein, we investigated whether a bioactive and degradable bioengineered micro-porous CTS encapsulated with ASC-derived exosomes could accelerate the healing process in diabetic rats.

## 2 Material and methods

All materials used in the present study were purchased from Sigma-Aldrich (St. Louis, MO), except where assigned otherwise.

### 2.1 Preparation and characterization of CTS

Processing and preparation of the CTS was carried out in the same manner as outlined before [9]. In short, skin samples were obtained from a healthy adult donor (aged 30–40 years) who had cosmetic mammoplasty or liposuction surgeries in a sterile environment and were immediately transported to the laboratory. The patient gave her consent after being informed for the use of her tissue. Before the decellularization process, the tissue samples were thawed, cut to dimensions of around 4 × 5 cm^2^, and thoroughly washed with distilled water multiple times to remove blood and other substances. Next, the outer layers of the skin were physically removed from the inner layer. Next, the components were immersed in trypsin/0.02% EDTA for an hour, followed by 3% triton for 3 h, and 4% sodium deoxycholic acid solution for 2 h. Lastly, the decellularized dermal matrix (dDM) was washed with sterile phosphate buffered saline (PBS) and then freeze-dried. In order to create the CTS, the dried dDM product was digested using 1% pepsin (W/W) and then mixed in 500 mM acetic acid for three days while being continuously stirred. Then, the final thick hydrogel solution was balanced to pH 7.4 with NaOH, transferred to 24-well plates, frozen at – 80 °C, and subsequently freeze-dried for 24 h.

To chemically crosslink the lyophilized scaffolds, we used 50 mM/L 2-(N-morpholino) ethanesulfonic in 70 % ethanol (pH 5.4), 30 mM/L 1-ethyl-3-(3-dimethylaminopropyl) carbodiimide (EDC), and 30 mM/L N-hydroxysulfosuccinimide (NHS). The resultant cross-linked CTSs were prepared with 1 mm in thickness and 15 mm in diameter, next lyophilized and stored at 4 °C until further use. DNA contents were analyzed to verify the elimination of cells and their remnants. We conducted a biodegradability test to assess the decomposition ability of CTS following interactions with biological elements.

Moreover, cell proliferation in dermal matrix (DM) samples was assessed using the MTT assay at various time points. In short, ASCs were placed into 24-well plates and incubated for 24 h in a 5% CO^2^ at 37 °C with 20 × 10^3^ cells per well. Afterwards, equal tissue samples were made from each group using a 0.6 cm biopsy punch, then weighed and placed in a 24-well plate. After making a 10 ml MTT solution (5 mg/ml), 100 μl (0.5 mg/ml in PBS) was added to each well, and the cells were then incubated at 37 °C for 4 h. The medium was removed and 600 μl dimethyl sulfoxide was added for 15 min. The spectrophotometer was used to measure the optical density (OD) values at a wavelength of 570 nm. Furthermore, the microstructure of the CTS was evaluated using scanning electron microscope (SEM). Finally, the average pore size was calculated from at least 10 measurements of SEM images using Image Analyzer software (Image J 1.44p).

### 2.2 Cell culture

The steps of collecting, culturing, and validating ASCs were performed according to our previous study [18]. Human subcutaneous adipose tissues from young women who had elective liposuction were collected. The patient gave consent after being properly informed for the use of her tissue. In short, after acquiring the adipose tissue, it was rinsed with PBS three times in order to remove blood and other substances. Next, the tissue fragments were treated with 0.1% collagenase type I for 50 min at ambient temperature. The cell pellet was isolated by centrifuging the solution at 800 rpm for 5 min. Afterwards, the cell pellets were mixed in Dulbecco's modified Eagle medium (DMEM; Gibco) supplemented with 20% fetal bovine serum (FBS; Gibco) and 1% penicillin-streptomycin (Gibco). Finally, the cells were placed in T-75 flasks and allowed to incubate.

### 2.3 Isolation and identification of ASCs-derived exosomes

ASCs-derived exosomes were isolated and identified following the protocol outlined by Khalatbary et al. [18]. In short, once the cells reached 70–80% confluence between passages 3–5, they were rinsed twice with sterile PBS and then placed in serum-free low glucose-DMEM (Gibco) for 48 h. Then, the liquid at the top was gathered, centrifuged at 3000 rpm for 20 min, and filtered through a 0.22 μm filter (Millipore) to eliminate cellular waste. Afterwards, 5 ml of sterile PBS was mixed with the isolated supernatant and then subjected to ultracentrifugation at 100 × 10^3^ rpm for 70 min. To conclude, the second phase of ultracentrifugation for exosome retrieval was replicated at a rate of 100,000 rpm for a duration of 70 min. The obtained exosomes were strained through a 0.22 μm pore filter and stored in a −80 freezer for additional analysis. Transmission electron microscope (TEM), dynamic light scattering (DLS), and western blotting were utilized to analyze the morphology, size, and typical exosome markers (CD9, CD63, and CD81), respectively [19].

### 2.4 Exosome loading on CTS and characterization

The sulfo-MBS was utilized for immobilizing ASCs-derived exosomes into some CTSs. To achieve this goal, the scaffolds were first soaked in a m-maleimidobenzoyl-N-hydroxysulfosuccinimide ester solution (sulfo-MBS; 1 mg/ml in PBS) for 1 h before being transferred into an exosomes solution (200 μg in 200 μl PBS) for an additional hour. The choice of the dose used was based on previous studies and our pilot study [18,20]. Ultimately, the CTS+Exo exosomes obtained were rinsed three times with sterile PBS for 1 h and then stored at −80 °C for future purposes.

The exosome release profile from the CTS was evaluated using a BCA protein assay kit (Takapouzist, Tehran, Iran) according to the manufacturer's instructions. Briefly, the CTS samples were placed in individual wells containing 1 ml of sterile PBS (pH 7.4) and incubated at 37 °C under static conditions to simulate physiological settings. At predetermined time intervals (0, 1, 3, 7, 14, and 21 days), 500 μl of the supernatant was collected from each well and immediately replaced with an equal volume of fresh PBS to maintain consistent experimental conditions. The collected supernatants were stored at −20 °C until further analysis. The concentration of released exosomes was quantified using the BCA protein assay, and the measurements were performed in triplicate to ensure reproducibility. Additionally, SEM was used to analyze the microstructure of CTS+Exo in order to verify the existence of exosomes.

### 2.5 Study design

A total of 60 healthy male Wistar rats were selected for the study. All animals were maintained in standard temperature, lighting, and provided with rodent chow and water. In all rats, Type 1 diabetes mellitus was induced by injecting (intraperitoneal) a single dose of 55 mg/kg of streptozotocin that was dissolved in 0.1 M citrate buffer (pH 4.5). After three days, fasting blood sugar levels were examined and classified as diabetic if they exceeded 250 mg/dl. All diabetic animals were kept for a period of thirty days in order to confirm the induction of diabetes [5,6]. The rats were allocated into 4 groups (n = 15) randomly: control group; CTS group with CTS engraftment; Exo group given 200 μg ASCs-derived exosomes in 200 μl PBS at four points around the wound (2 mm from edges); and CTS+Exo group with CTS preloaded with 200 μg ASCs-derived exosomes. Sampling was conducted on three occasions - on the 7th, 14th, and 21st days post wound creation, with 5 samples collected from each group at each time point for wound tissue assessment [5,7].

### 2.6 Surgery

To wound creation, the rats were positioned in a prone and had the hair on their chest's back removed. Following the use of 70% ethanol to clean the area, a circular wound with a diameter of 15 mm was made. To alleviate pain, all animals received ibuprofen (20 mg/kg) every 8–12 h before and up to five days following surgery.

To create a delayed wound, a circular ring was used, which was sutured around the wound and prevented spontaneous contraction of the wound [21]. Also, in order to create an ischemic model, two longitudinal wounds (10 cm long) were created on either side of the wound, cutting off the blood supply to the wound site [5]. Using a digital camera (FinePix S20, Japan), the wound size progress was captured on days 7, 14, and 21 post-surgery. Next, the images were examined with MacBiophotonics in ImageJ software (National Institutes of Health) and the rate of wound closure was measured and compared between the experimental groups at each specific time, using a specific formula:

$$\text{Wound}\;\text{closure}\;\text{rate}\;\left(\%\right)=\frac{A_{0}-A_{n}}{A_{0}}\times 100$$

A_n_: wound size on day n; and A_0_: wound size on day 0.

### 2.7 Histological and stereological assessments

The entire thickness of the wound tissues and surrounding healthy skin collected and preserved in 10% formalin. Following standard histological protocols, the samples were encased in paraffin. Then, ten equally spaced segments were chosen from every rat and stained using hematoxylin and eosin (H&E). Additionally, masson's trichrome (MT) staining was conducted to assess collagen density in the newly formed dermis.

To assess the volumes of newly formed epidermis and dermis at the wound site, the following formula was used:

$$V_{total}=\sum P\times \frac{a}{p}\times t$$

ΣP: total counted points; $\frac{a\;\left(area\right)}{p\;\left(point\right)}$; t: thickness.

The formula below was utilized to assess the numerical densities (Nv) of fibroblasts, neutrophils, and blood vessels:

$$N_{v}=\frac{\sum Q}{\sum P\times h\times \frac{a}{f}}\times \frac{t}{BA}$$

ΣQ: represents the cell number; h: represents the height of the dissector; Σp: represents the total number of cells counted within the probe; $\frac{a\;\left(area\right)}{f\;\left(field\right)}$: represents the area per field; BA: represents the block advance of the microtome; and t: represents the section thickness.

In addition, the formula below was utilized to evaluate blood vessel density:

$$L_{v}=\frac{2\sum Q}{\sum P\times \frac{a}{f}}$$

ΣQ: represents the overall count of blood vessels; Σp: represents the total counted points; $\frac{a\;\left(area\right)}{f\;\left(field\right)}$.

### 2.8 Gene expression analysis

At present, we have assessed the expression levels of transforming growth factor beta (TGF-β) and basic fibroblast growth factor (bFGF) genes related to proliferation and regeneration, vascular endothelial growth factor (VEGF) gene related to angiogenesis, microRNA-146a (miRNA-146a) gene related to inflammation regulation, and tumor necrosis factor alpha (TNF-α) and interleukin-1 beta (IL-1β) genes related to inflammation using quantitative real-time PCR (qRT-PCR) on day 7. To achieve this goal, the complete RNA was collected with the RNeasy Mini Kit (Qiagen). Afterwards, cDNA synthesis was performed with iScript™ cDNA Synthesis Kit (Bio-Rad) [18]. Real-time PCR were performed with a Bio-Rad instrument. Table 1 displays the primers sequences that were utilized. Primer efficiency was assessed by constructing a standard curve for each gene, and efficiency values were calculated based on the slope of the curve. Efficiency values between 90 % and 110 % were considered acceptable. Furthermore, primer specificity was confirmed by melting curve analysis, where a single peak indicated the amplification of the target gene with no non-specific products. In addition, validation of gene expression data was carried out using β-actin as an internal control, and relative gene expression levels were calculated using the Livak method.

**Table 1:** Primer sequences used for gene expression analysis using quantitative RT-PCR.

Gene	Sequence (5 ' > 3 ′)	Reference
miRNA-146a	**F:** CTGAGAACTGAATTCCATGGGT **R:** ATGACGATAGAGCTATCCCAGC	Ghasemi et al. [28]
TGF-β	**F:** CTGAACCAAGGAGACGGAAT **R:** GGTTCATGTCATGGATGGTG	Savari et al. [44]
bFGF	**F:** GTCAAACTACAGCTCCAAGC **R:** TTTATACTGCCCAGTTCGTT	Elbialy et al. [45]
VEGF	**F:** AGGCTGCACCCACGACAGAA **R:** CTTTGGTCTGCATTCACATC	Elbialy et al. [45]
TNF-α	**F:** CCCAGACCCTCACACTCAGAT **R:** TTGTCCCTTGAAGAGAACCTG	Kandhare et al. [46]
IL-1β	**F:** TGGCAGCTACCTATGTCTTGC **R:** CCACTTGTTGGCTTATGTTCTG	Omran et al. [47]
Β-actin	**F:** GAGAGGGAAATCGTGCGTGAC **R:** CATCTGCTGGAAGGTGGACA	Omran et al. [47]

### 2.9 Tensiometery

Tissue samples measuring 5 × 0.5 cm were collected on the 21st day from five rats in each group and mounted on a material testing machine (Santam Co). The elongation rate was maintained at 0.166 mm/s while identifying the maximum force (in NEWTON), energy absorption (in JOULE), bending stiffness (in MPa), and stress at high load (in NEWTON/Cm^2^) based on the load-elongation curves.

### 2.10 Statistical analysis

The data was examined using SPSS software (IBM SPSS Statistics, version 23, IBM, Armonk, NY, USA). An ANOVA test was conducted followed by Tukey's post hoc test to assess the associations among different groups. The quantitative data was presented as the mean ± standard deviation (SD) and the p-values less than 0.05 considered significant.

## 3 Results

### 3.1 Characteristics of dDM/CTS

**Fig. 1:**
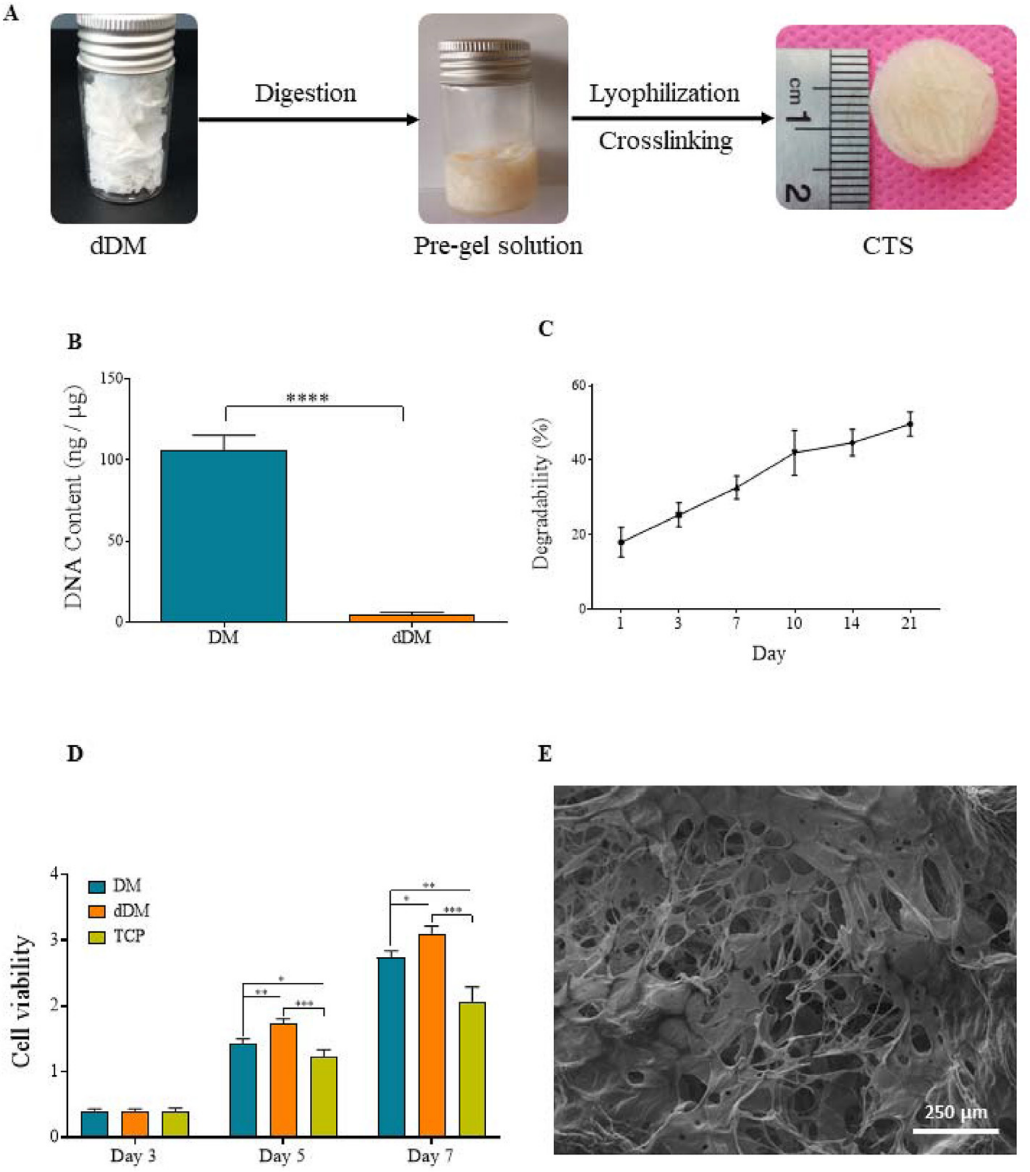
**Characterization of CTS.** (A) Flowchart from dermal matrix decellularization to CTS production. (B) Quantification of DNA amount confirmed the quality of the decellularization method. (C) The biodegradability of CTS is shown using enzymatic degradation. (D) The MTT photograph showed that there was a significant difference between the experimental groups and control group after 7 days. These results suggested that the CTSs promoted the ASCs proliferation and had no cytotoxic effects (Data are represented in Mean ± SD; ∗p < 0.05, ∗∗p < 0.01, ∗∗∗p < 0.001, ∗∗∗∗p < 0.0001). (E) Scanning electron micrographs of the CTS shows the micro-porous structure (magnification: 80 X).

Determination of DNA content showed that the quantity of DNA decreased to below 4 ng/μg, suggesting that over 95% of the cellular DNA was eliminated from the dDM (Fig. 1B). Evaluating enzymatic degradation in CTS indicated that the biodegradability of the CTS reached about 49% on day 21 in a time dependent manner (Fig. 1C). In addition, cell proliferation was greater in both tissue samples than in tissue culture plates (TCP) at all-time points (Fig. 1D). Nevertheless, the dDM group showed the most favorable outcome on days 5 and 7, displaying a minor growth in cell count when compared to the intact dermis sample. SEM analysis indicated that CTS possesses a highly porous ultrastructure, with an average pore size of about 80 μm, making it suitable for cell migration, homing, and angiogenesis (Fig. 1E).

### 3.2 Characterization of ASCs-derived exosomes

**Fig. 2:**
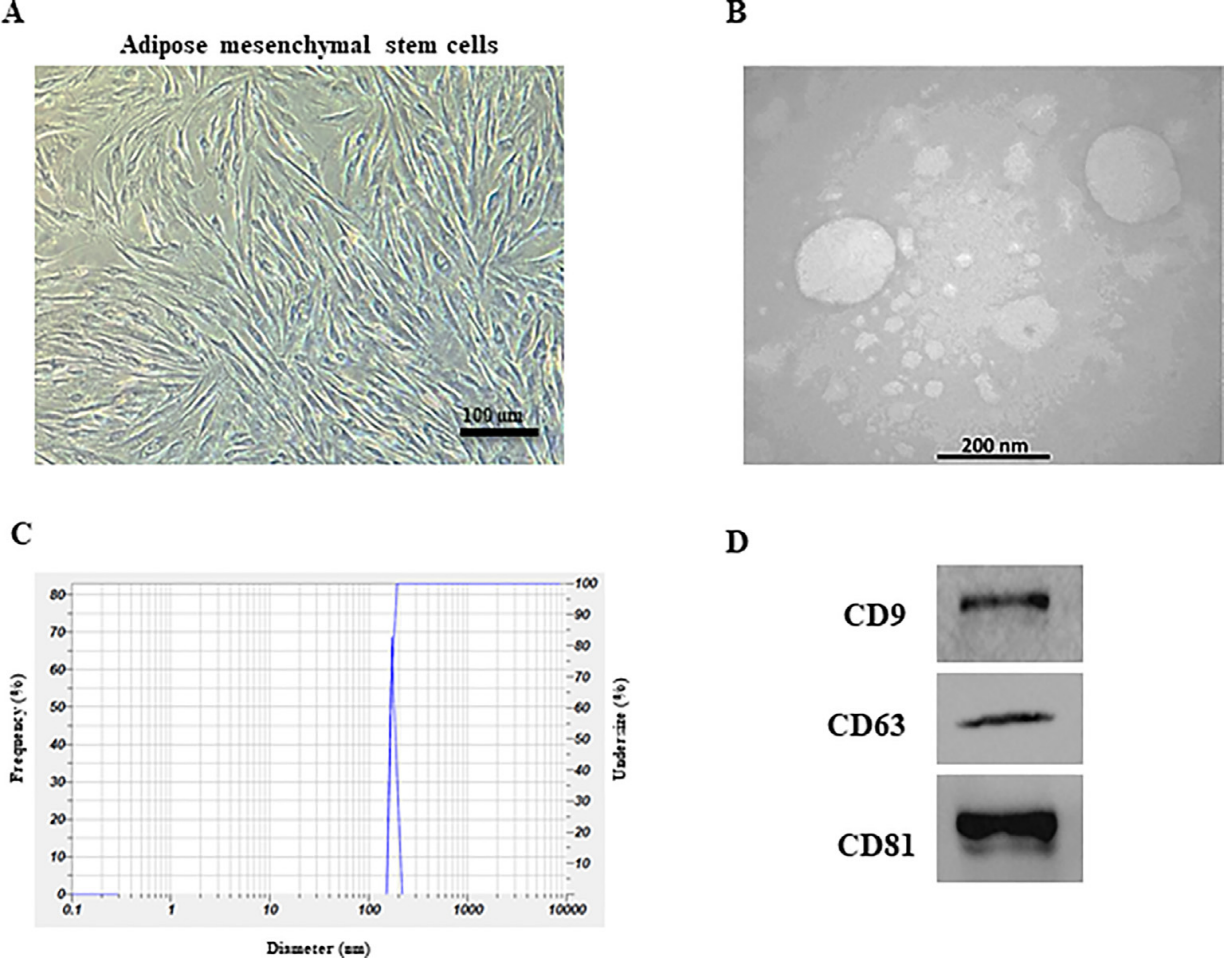
**Identification of ASCs and ASC- derived exosomes.** (A) ASCs grew as spindle-shaped, fibroblast cell colonies. (B) Morphology of ASCs-derived exosomes obtained by TEM. (C) The distribution of ASCs-derived exosomes size by DLS analysis. (D) Exosome specific surface proteins (CD9, CD63, and CD81) were examined by Western blot analysis.

As shown in Fig. 2A, ASCs grew as spindle-shaped, fibroblast cell colonies. The TEM image depicted round exosomes that were obtained (Fig. 2B). The average exosome diameter measured 167.6 ± 9.7 nm (Fig. 2C). Furthermore, Western blot analysis indicated that the presence of exosome surface proteins CD9, CD63, and CD81 (Fig. 2D).

### 3.3 Characteristics of DMS+Exo

**Fig. 3:**
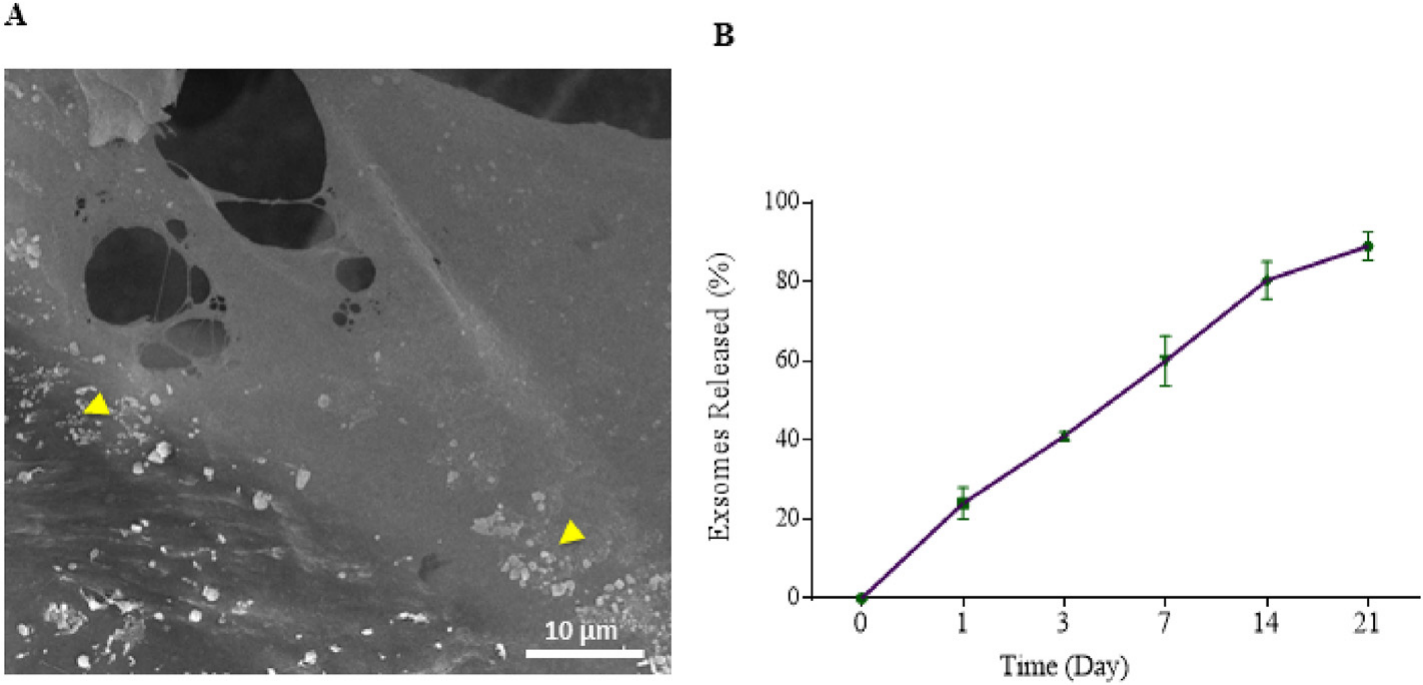
**Characterization of CTS+Exo.** (A) SEM shows the micro-porous structure of the CTS that the exosomes were distributed in the pores (arrows head) (magnification: 2000 X). (B) The levels of exosomes release from the CTS was evaluated using a BCA protein assay kit at predetermined time points, day 0, 1, 3, 7, 14, and 21.

Examining the ultrastructure by SEM, we noted a consistent spread of exosomes within the scaffold (Fig. 3A). In addition, the CTS exhibited a profile of releasing exosomes (24 ± 4 % on day 1–89 ± 3.6 % on day 21), demonstrating efficient encapsulation within the CTSs, resulting in sustained long-term release of exosomes (Fig. 3B).

### 3.4 Wound closure rate

**Fig. 4:**
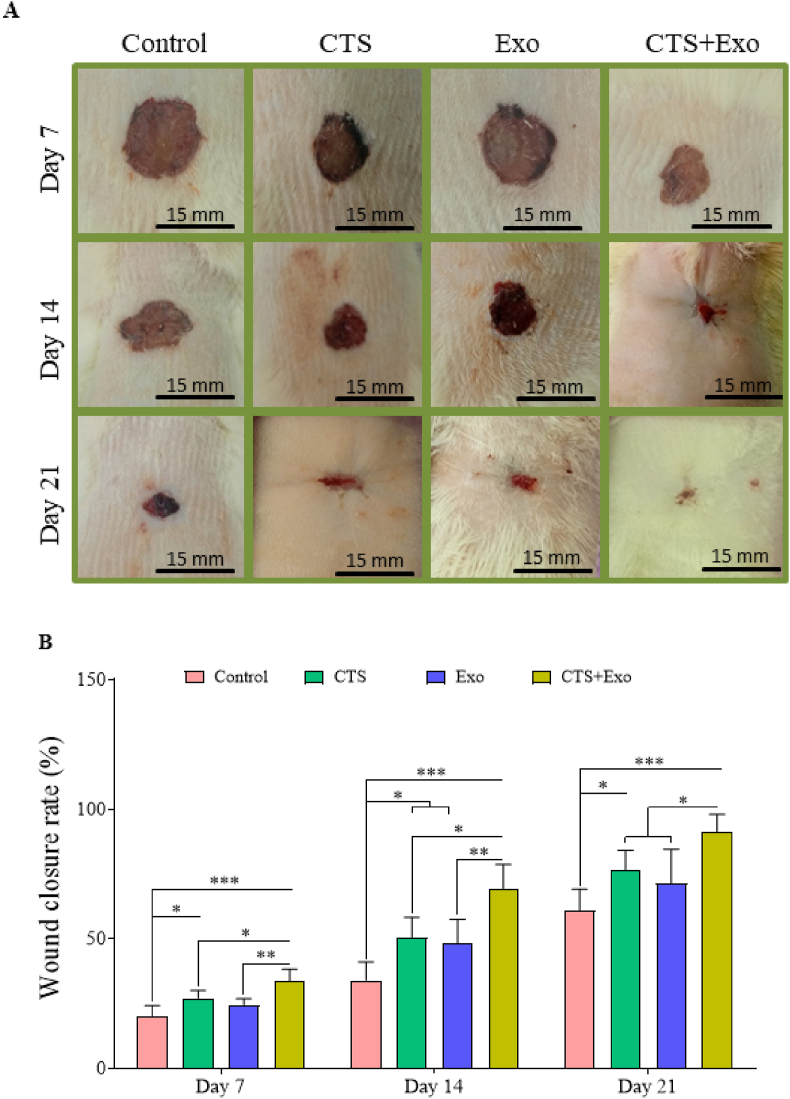
**The impact of CTS transplantation in combination with ASCs-derived exosomes on wound closure rate.** (A) The photographs represent progressive healing of the wounds over time, to compare the four experimental groups. (B) The percentage of wound closure rate during the study period concerning the initial wound area. Data are presented as Mean ± SD. An ANOVA test was conducted followed by Tukey's post hoc test to assess the associations among different groups. Statistical significance is indicated by ∗p < 0.05, ∗∗p < 0.01, ∗∗∗p < 0.001, ∗∗∗∗p < 0.0001, showing significant differences between the groups.

The photographs from the wound area at three-time points are shown in Fig. 4A. Analysis of wound contraction rate showed that the wound size notably reduced in the CTS and CTS+Exo groups than the control group on days 7 (p = 0.034 and p = 0.000), 14 (p = 0.032 and p = 0.000), and 21 (p = 0.025 and p = 0.000). Additionally, it was discovered that the Exo group exhibited a substantially greater rate of wound closure than the control group on day 14 (p = 0.037). Moreover, results indicated that the wound size in the CTS+Exo group was considerably smaller compared to the CST and Exo groups on days 7 (p = 0.05 and p = 0.005), 14 (p = 0.016 and p = 0.006), and 21 (p = 0.041 and p = 0.018) (Fig. 4B).

### 3.5 Histological and stereological findings

**Fig. 5:**
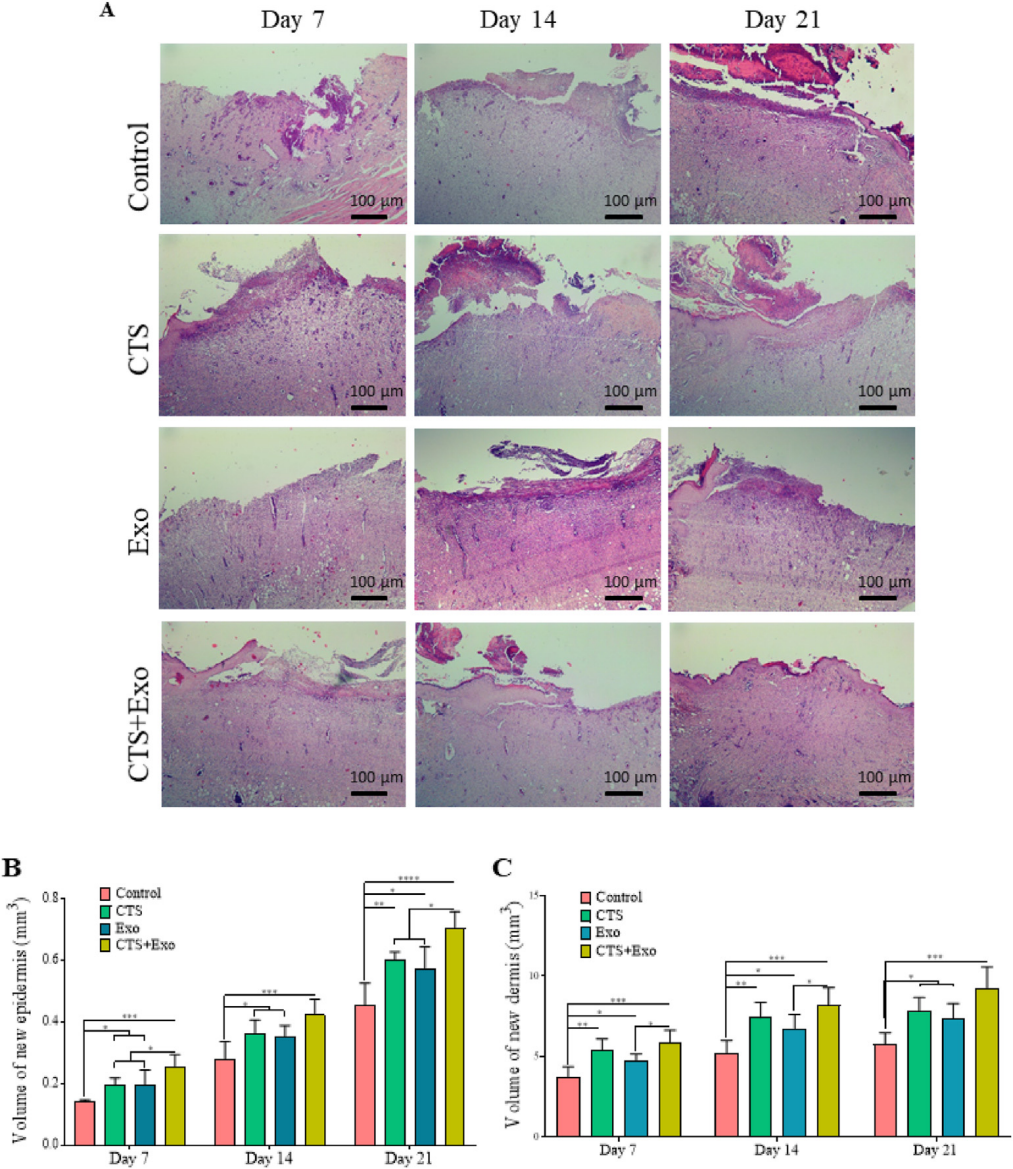
**The impact of CTS transplantation in combination with ASCs-derived exosomes on histological and stereological changes.** (A) The photomicrographs of H&E staining from the new epidermis and dermis on days 7, 14, and 21. (B) Volumes of the new epidermis and (C) dermis in the healing wounds were calculated by Cavalier's method. Data are presented as Mean ± SD. An ANOVA test was conducted followed by Tukey's post hoc test to assess the associations among different groups. Statistical significance is indicated by ∗p < 0.05, ∗∗p < 0.01, ∗∗∗p < 0.001, ∗∗∗∗p < 0.0001, showing significant differences between the groups.

**Fig. 6:**
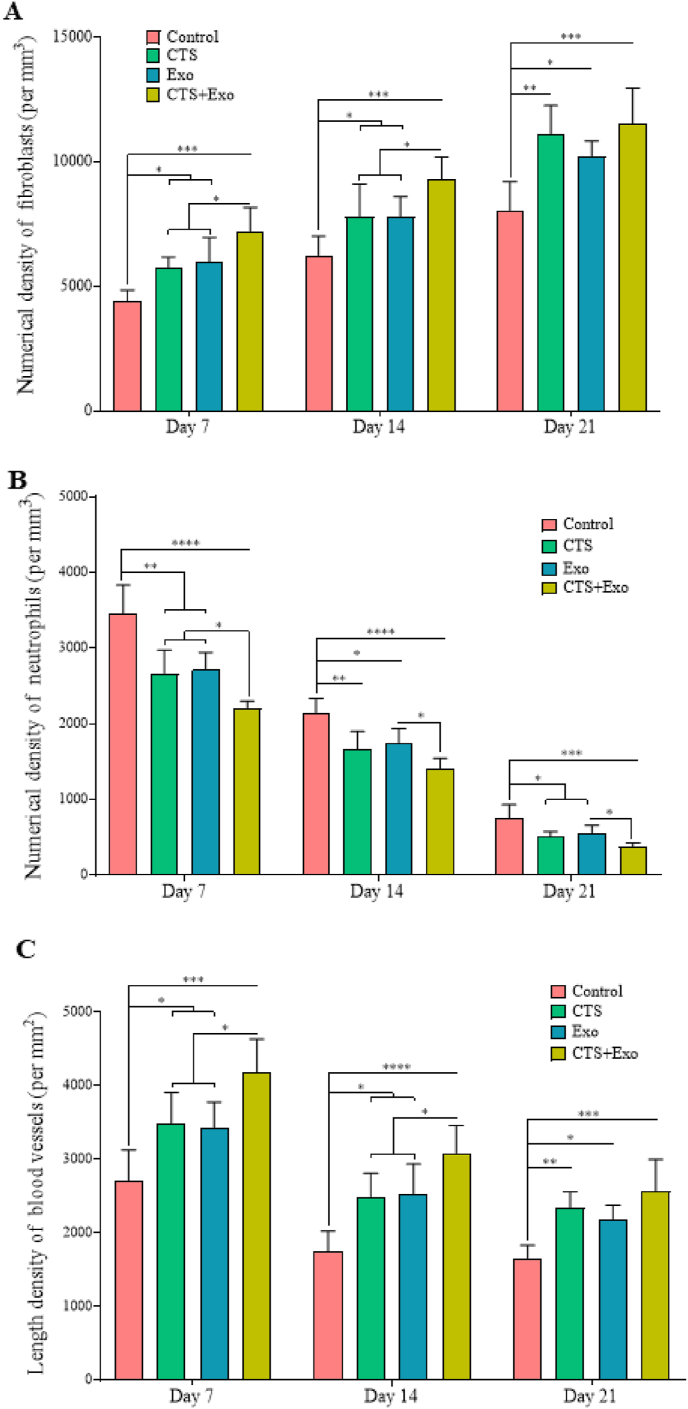
**The impact of CTS transplantation in combination with ASCs-derived exosomes on stereological parameters.** Numerical densities of (A) fibroblasts, (B) neutrophils, and (C) blood vessels in the wound bed on days 7, 14, and 21. Data are presented as Mean ± SD. An ANOVA test was conducted followed by Tukey's post hoc test to assess the associations among different groups. Statistical significance is indicated by ∗p < 0.05, ∗∗p < 0.01, ∗∗∗p < 0.001, ∗∗∗∗p < 0.0001, showing significant differences between the groups.

Fig. 5A displays tissue sections that have been stained with H&E. The quantitative data for stereological examinations can be found in Fig. 5, Fig. 6C.

#### 3.5.1 Volume of new epidermis and new dermis

The findings indicated that the CTS, Exo, and CTS+Exo groups had significantly increased new epidermis volume compared to the control group on days 7 (p = 0.044, p = 0.032, and p = 0.000), 14 (p = 0.048, p = 0.039, and p = 0.000), and 21 (p = 0.005, p = 0.029, and p < 0.000). Additionally, the comparison of results among treatment groups showed that the CTS+Exo group exhibited a considerably higher amount of new epidermis in comparison to the CTS and Exo groups on days 7 (p = 0.044, p = 0.041) and 21 (p = 0.033, p = 0.012) (Fig. 5B).

Considering the new dermis volume, significantly higher dermis volumes were noticed in the CTS, Exo, and CTS+Exo groups compared to the control group on days 7 (p = 0.003, p = 0.046, and p < 0.000), 14 (p = 0.01, p = 0.49, and p = 0.000), and 21 (p = 0.022, p = 0.045, and p = 0.000). Moreover, the volume of the new dermis in the CTS+Exo group showed a significant increase compared to the Exo group on days 7 and 14 (both, p = 0.046) (Fig. 5C).

#### 3.5.2 The numerical density of fibroblasts

In terms of fibroblast numbers, significantly higher values were found in the CTS, Exo, and CTS+Exo groups on days 7 (p = 0.048, p = 0.025, and p = 0.000), 14 (p = 0.04, p = 0.042, and p = 0.000), and 21 (p = 0.002, p = 0.037, and p = 0.000) compared to the control group. Additionally, the CTS+Exo group shows significantly higher fibroblasts compared to the CTS and Exo groups, on days 7 (p = 0.031 and p = 0.042) and 14 (p = 0.049 and p = 0.048) (Fig. 6A).

#### 3.5.3 The numerical density of neutrophils

When examining the number of neutrophils, we noted that the CTS, Exo, and CTS+Exo groups had significantly fewer cells than the control group on days 7 (p = 0.002, p = 0.000, and p < 0.000), 14 (p = 0.005, p = 0.023, and p < 0.000), and 21 (p = 0.015, p = 0.044, and p = 0.000). Additionally, the number of neutrophils in the CTS+Exo group was significantly reduced compared to the Exo group on days 7, 14, and 21 (p = 0.05). Furthermore, the CTS+Exo group showed a notable decrease in neutrophils compared to the CTS group on day 7 (p = 0.047, p = 0.05, and p = 0.43) (Fig. 6B).

#### 3.5.4 The length density of blood vessels

There was a considerable increase in the number of blood vessels in the CTS, Exo, and CTS+Exo groups than the control group on days 7 (p = 0.047, p = 0.043, and p = 0.000), 14 (p = 0.022, p = 0.016, and p = 0.000), and 21 p = 0.007, p = 0.044, and p = 0.000). Additionally, the comparison of outcomes among the treatment groups showed that the CTS+Exo group had a higher number of blood vessels than the CTS and Exo groups on both days 7 (p = 0.048 and p = 0.05) and 14 (both, p = 0.05) (Fig. 6C).

### 3.6 Collagen deposition

**Fig. 7:**
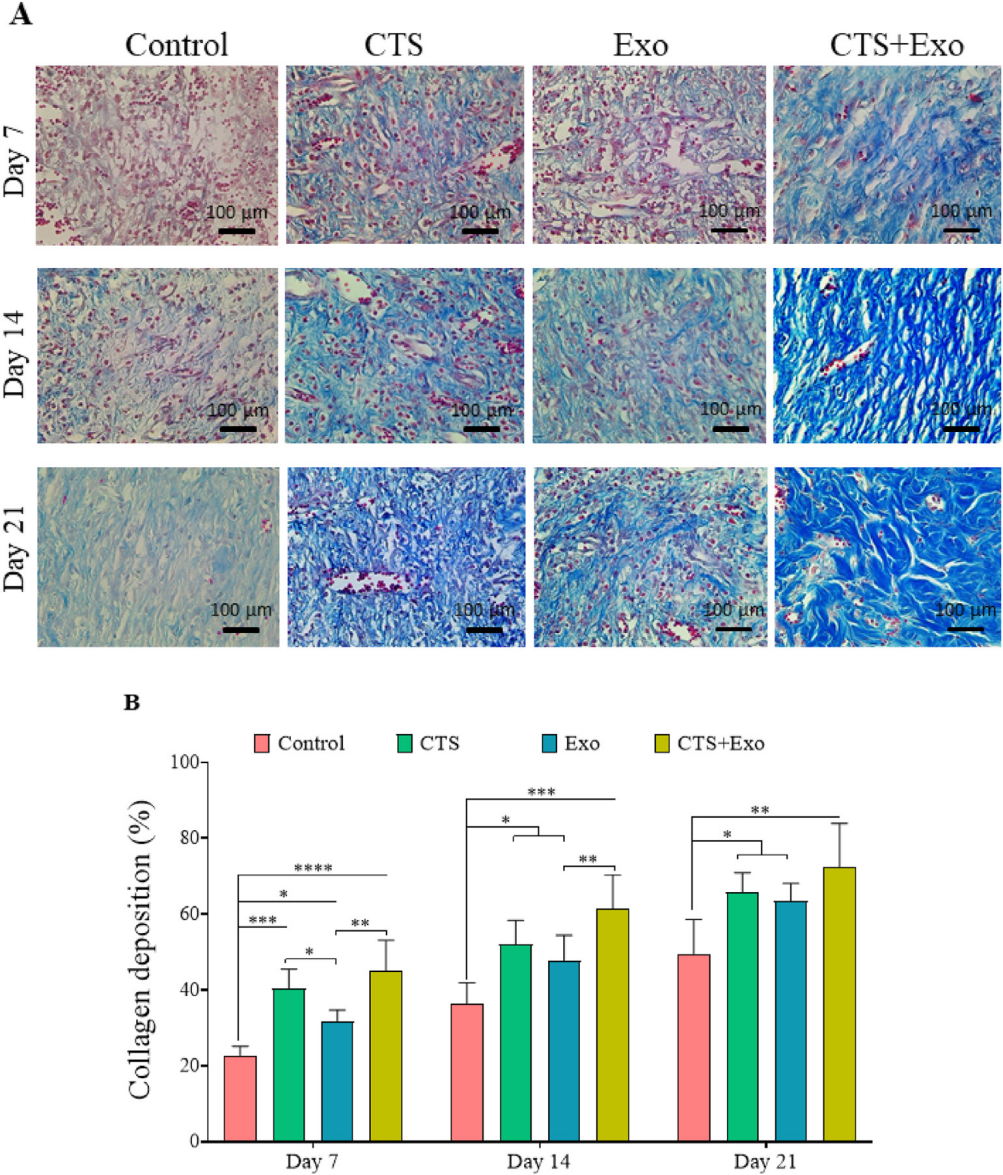
**The impact of CTS transplantation in combination with ASCs-derived exosomes on collagen deposition.** (A) Representative micrographs of the newly formed dermis stained by Mallory's trichrome methods to show collagen in blue color. (B) The quantitative analysis of the amount of collagen density. Data are presented as Mean ± SD. An ANOVA test was conducted followed by Tukey's post hoc test to assess the associations among different groups. Statistical significance is indicated by ∗p < 0.05, ∗∗p < 0.01, ∗∗∗p < 0.001, ∗∗∗∗p < 0.0001, showing significant differences between the groups.

MT staining was conducted to assess the level of collagen production and accumulation in the new dermis (Fig. 7A). It was found that collagen deposition significantly rose in the CTS, Exo, and CTS+Exo groups in comparison with control group on days 7 (p = 0.000, p = 0.05, and p < 0.000), 14 (p = 0.012, p = 0.05, and p = 0.000), and 21 (p = 0.026, p = 0.05, and p = 0.001). Additionally, we noticed that the amount of collagen deposited in the CTS+Exo group was significantly higher than in the Exo group, on days 7 and 14 (p = 0.005 and p = 0.034). Moreover, the CTS group exhibited significantly greater collagen density than the Exo group on day 7 (p = 0.05) (Fig. 7B).

### 3.7 Gene expression analysis

**Fig. 8:**
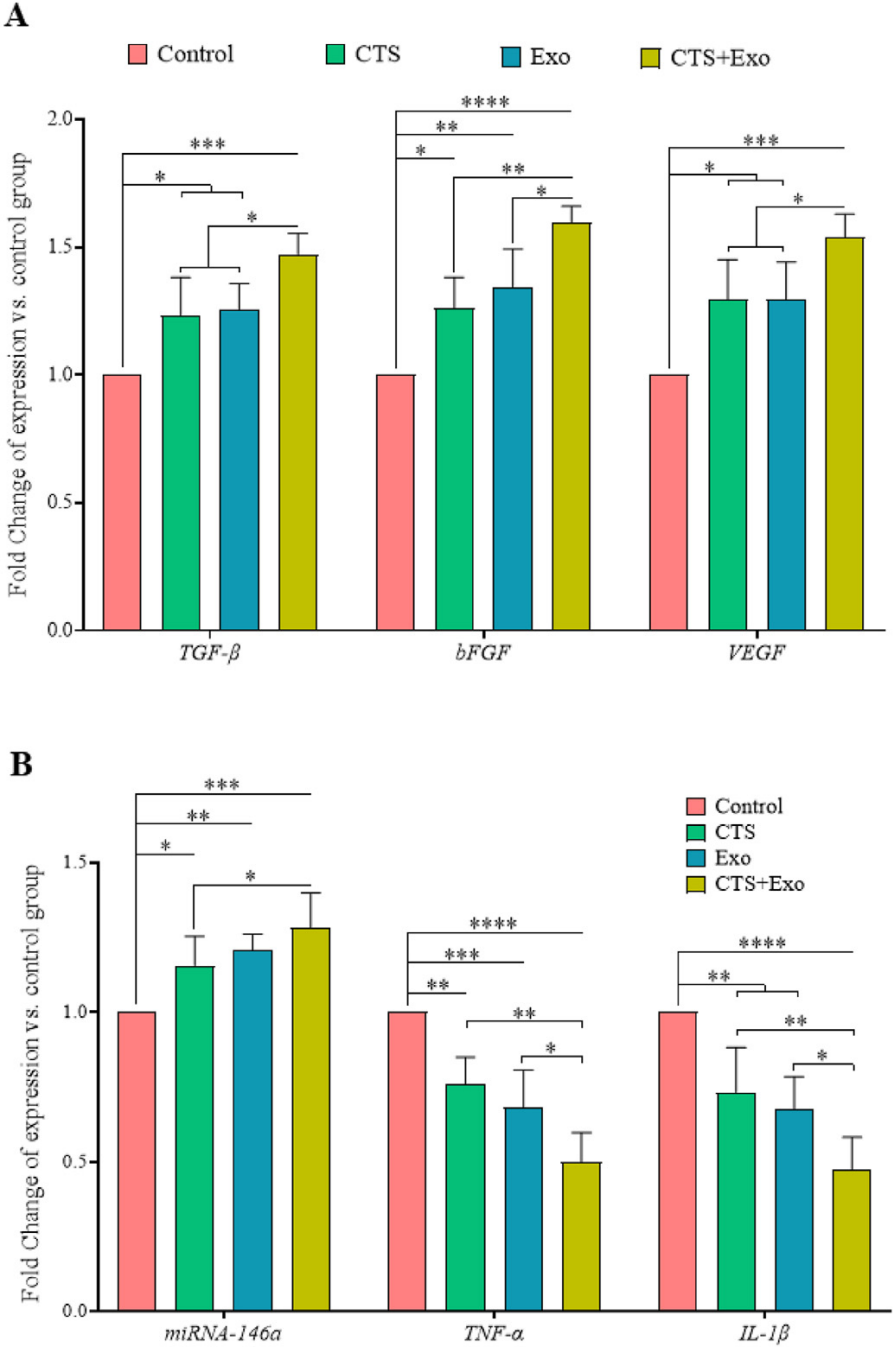
**The impact of CTS transplantation in combination with ASCs-derived exosomes on gene expression.** The expression levels for (A) TGF-β, bFGF, and VEGF (contributing to regeneration and angiogenesis), (B) miRNA-146a (involved in reducing and regulating inflammation), and TNF-α and IL-β (involved in inflammation) were determined on day 7, using qRT-PCR. Relative expression levels were normalized to β-actin and calculated using the Livak method. Data are presented as Mean ± SD. An ANOVA test was conducted followed by Tukey's post hoc test to assess the associations among different groups. Statistical significance is indicated by ∗p < 0.05, ∗∗p < 0.01, ∗∗∗p < 0.001, ∗∗∗∗p < 0.0001, showing significant differences between the groups.

The levels of TGF-β, bFGF, and VEGF genes were notably increased in the CTS, Exo, and CTS+Exo groups compared to the control group when evaluating gene expression related to regeneration and angiogenesis (p = 0.046, p = 0.026, and p = 0.000 for TGF-β; p = 0.015, p = 0.001, and p < 0.000 for bFGF; and p = 0.028, p = 0.029, and p = 0.000 for VEGF). Moreover, when comparing the CTS+Exo group to the CTS and Exo groups, significant enhancements were observed for TGF-β (p = 0.042 and p = 0.05), bFGF (p = 0.002 and p = 0.022), and VEGF (both, p = 0.05) genes (Fig. 8A).

Regarding the expression levels of miRNA-146a, as an anti-inflammatory gene, we observed that the CTS, Exo, and CTS+Exo groups remarkably upregulated than the control group (p = 0.05032 p = 0.003, and p = 0.000). Furthermore, the CTS+Exo group had notably higher expression levels of miRNA-146a gen than the CTS group (p = 0.05) (Fig. 8B).

Regarding inflammatory genes, we found that in comparison with the control group, dramatically downregulation was occurred in the CTS, Exo, and CTS+Exo groups for TNF-α (p = 0.003, p = 0.000, and p < 0.000) and IL-1β (p = 0.005, p = 0.001, and p < 0.000) genes. Furthermore, comparing the results between treated groups showed that the CTS+Exo group compared to the CTS and Exo groups had notably lower expression of TNF-α (p = 0.001 and p = 0.028) and IL-1β (p = 0.007 and p = 0.039) genes (Fig. 8B).

### 3.8 Biomechanical characteristics

**Fig. 9:**
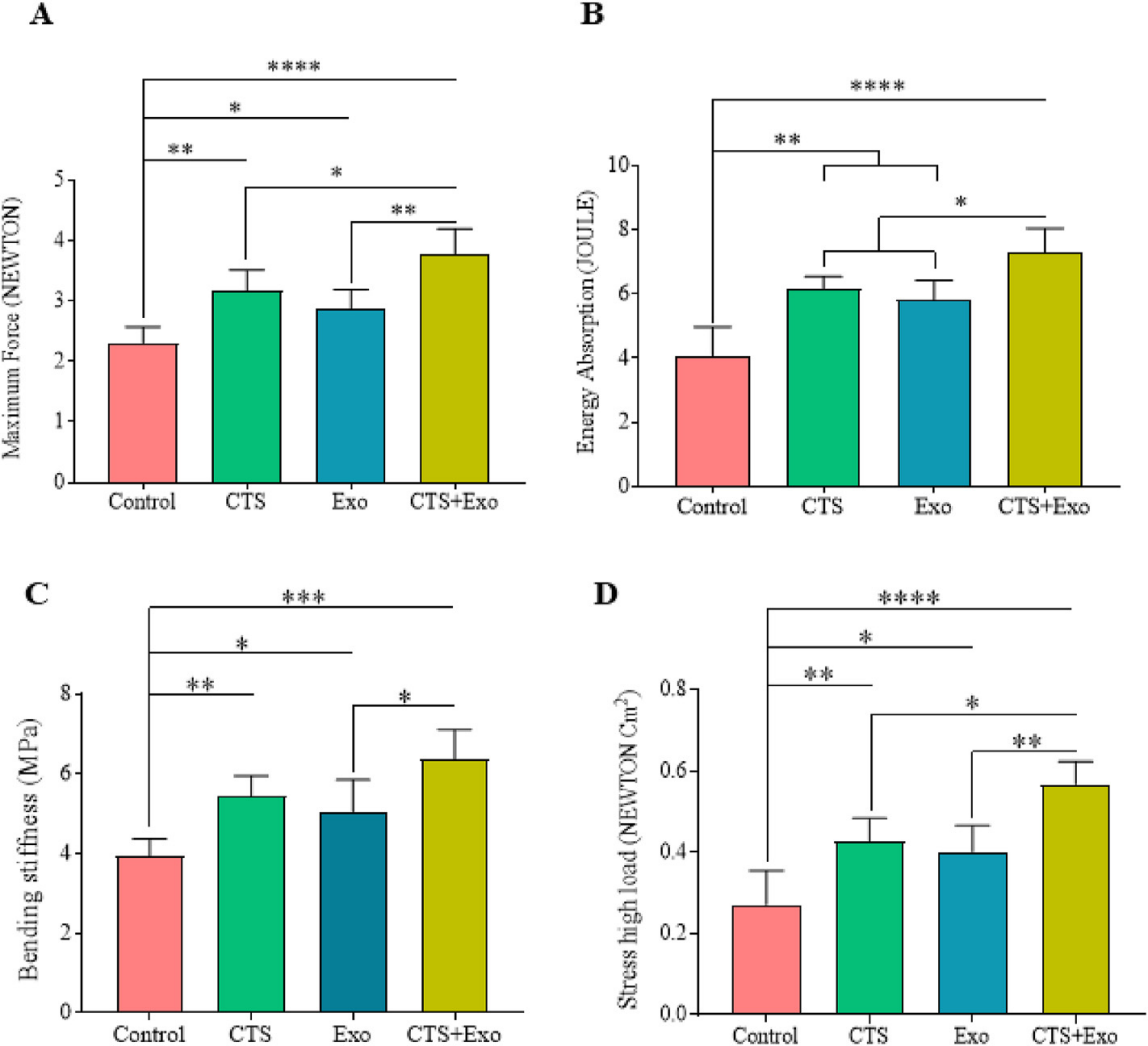
**The impact of CTS transplantation in combination with ASCs-derived exosomes on biomechanical parameters.** Biomechanical parameters were assessed on day 21. Data are presented as Mean ± SD. An ANOVA test was conducted followed by Tukey's post hoc test to assess the associations among different groups. Statistical significance is indicated by ∗p < 0.05, ∗∗p < 0.01, ∗∗∗p < 0.001, ∗∗∗∗p < 0.0001, showing significant differences between the groups.

The outcomes showed that the CTS, Exo, and CTS+Exo groups displayed significantly greater maximum force (p = 0.004, p = 0.05, and p < 0.000), energy absorption (p = 0.001, p = 0.004, and p < 0.000), bending stiffness (p = 0.009, p = 0.05, and p = 0.000), and stress high load (p = 0.008, p = 0.029, and p < 0.000) compared to the control group. Additionally, it was found that the CTS+Exo group had higher maximum force, energy absorption, and stress high load compared to the CTS (p = 0.05, p = 0.05, and p = 0.017) and Exo (p = 0.003, p = 0.016, and p = 0.005) groups. Furthermore, regarding the comparing of the bending stiffness parameter between treatment groups, the results showed that the CTS+Exo group had significantly higher score compared to the Exo group (p = 0.028) (Fig. 9).

## 4 Discussion

Due to their complex nature, diabetic wounds necessitate treatments that address multiple factors [3]. In this study, the impact of using bioactive and degradable bioengineered CTS containing ASCs-derived exosomes was assessed in delayed and ischemic wounds. We conducted assessments at three different time points to study how well the treatments work in healing diabetic wounds. Day 7 marks the peak of the inflammatory phase and the start of the proliferative phase, day 14 signifies the peak of the proliferative phase and the beginning of the regeneration phase, and day 21 represents the peak of the regeneration phase [5,7].

An important aspect of the scaffold's performance is its biodegradability, which is crucial for its long-term functionality and therapeutic effectiveness in diabetic wound healing [22]. In our study, we observed a 49 % biodegradability of the CTS at day 21, which indicates a controlled and gradual degradation process. This level of biodegradation is significant because it allows the scaffold to provide sustained structural support during the early and middle phases of wound healing, especially when cellular activities such as migration, proliferation, and angiogenesis are most active. By day 21, the scaffold's partial degradation ensures that it does not interfere with the final stages of tissue remodeling and wound closure, promoting a smooth transition to fully regenerated tissue. The controlled biodegradability of the CTS is also essential for the sustained release of exosomes. As the scaffold degrades over time, it continuously releases exosomes in a time-dependent manner, which helps maintain a steady supply of regenerative factors at the wound site. This sustained release is particularly beneficial for chronic diabetic wounds, where cellular function is often impaired, and tissue repair is slow. The gradual degradation of the scaffold, combined with the release of exosomes, supports a prolonged therapeutic effect, which is critical for maintaining long-term wound healing and tissue regeneration.

Furthermore, analysis of CTS loaded with ASCs-derived exosomes demonstrate that the exosome release followed a sustained, linear release mechanism over time, suggesting a controlled release driven by the biodegradation of the CTS. Regarding the in vivo results of the present study, the sustained and controlled release of exosomes from the CTS plays a significant role in promoting tissue regeneration and enhancing wound healing, particularly in the context of diabetic wounds. Diabetic patients often experience chronic, non-healing wounds due to impaired cellular function, prolonged inflammation, and defective angiogenesis. In this regard, the ability of the CTS to release exosomes in a time-dependent and controlled manner provides a promising strategy for addressing these challenges. The slow and continuous release of exosomes over a period of 21 days, as shown in our study, ensures a prolonged therapeutic effect. This sustained release profile is beneficial for maintaining a steady supply of regenerative factors, which is essential for stimulating cellular activities such as migration, proliferation, and angiogenesis at the wound site.

In wound tissue evaluations, we generally found that the use of CTS loaded with ASCs-derived exosomes considerably increase the wound closure rate and collagen density, improve the stereological and biomechanical characterizes, and upregulated the regenerative and angiogenesis cytokines. Furthermore, we observed a simultaneous decrease in the numerical density of neutrophils and inflammatory cytokines in treatment groups, with the most significant changes seen in the CTS+Exo group.

In the process of wound healing at the molecular and cellular levels, the first phase is the inflammatory stage, posing a significant challenge to healing in diabetic wounds due to its prolonged duration [23]. To assess the inflammation at the wound site, we employed two techniques: stereological assessment of neutrophil density and analyzing gene expression levels related to inflammation, including miRNA-146a which helps reduce and regulate inflammation, along with two other inflammatory genes, TNF-α and IL-1β. We noticed that the treated groups indicated notable changes in all inflammatory markers than the control group, with the most noticeable differences seen in the CTS+Exo group. In this regard, Milan et al. found that hydrogel from dermis matrix has anti-inflammatory properties and can reduce local inflammation during wound healing [7]. Yang et al. examined how combining dermis matrix and MSCs affects the process of acute wound healing. According to their findings, the utilized matrix effectively reduced the release of inflammatory cytokines like TNF-*α* and IL-6 [24]. Furthermore, Bour et al. found that the dermis-based scaffold includes anti-inflammatory elements which, when applied to diabetic wounds, can notably lessen the levels of inflammatory genes and the presence of neutrophils in the area [9].

However, Davoodi et al. as well as Bour et al. reported that ASCs induce the production of certain anti-inflammatory cytokines like TGF-β, leading to the regulation of inflammation at the wound site [11,13]. Li et al. reported that exosomes derived from ASCs exhibit strong anti-inflammatory properties and are able to greatly reduce the levels of TNF-α and IL-1β genes at the site of injury. As stated by them, the effects are believed to be caused by the increased levels of Nrf2 that inhibits lipid peroxidation and the activation of pro-inflammatory cytokines [16]. In addition, Blazquez et al. found that exosomes from ASCs inhibit T-cell activation by decreasing IFN-γ secretion and do not contain MHC class II and costimulatory molecules, suggesting a direct inhibitory effect on T cell activation [25].

Additionally, we assessed the mRNA levels of the miRNA-146a gene in the different experimental groups and observed a notable increase in its expression levels in the treated groups, particularly the CTS+Exo group, in comparison to the control group. EI Gazzar et al. found that miR-146a led to a reduction in acute inflammatory cytokine expression via a signaling pathway [26]. Feng et al. and Moravej et al. found a correlation between higher miRNA-146a levels and lower IL-1β expression at the wound location [27,28]. As a result, we propose that miRNA-146a could have inhibited IL-1β expression in this study. Therefore, we determined that utilizing CTS and ASCs-derived exosomes simultaneously had a synergistic effect in reducing inflammation.

The second phase of wound healing is known as proliferation. In the proliferative phase, there was growth in the volumes of the new epidermis and dermis, an increase in fibroblast numbers, and higher levels of gene expression related to proliferation [6,11,29]. Nonetheless, in wounds of diabetic patients, the normal progression of raising these factors is disrupted by oxidative stress and inflammation [5,30]. Currently, we assessed the proliferative phase by examining the volumes of the new epidermis and dermis, as well as the quantities of fibroblasts in the new dermis through stereological analysis. Additionally, we measured the levels of TGF-β and bFGF using molecular techniques. A notable rise was noticed in all factors within the treated groups, particularly in the CTS+Exo group in comparison with the control group. Yu et al. found that the matrix derived from dermis included a large number of cytokines, which influenced cell growth and promoting the healing of wounds [31]. This aligns with the findings of Bondioli et al. who showed that porous three-dimensional structures with biological ligands can greatly enhance attachment, growth, movement, and differentiation of the cells [32]. However, Li et al. found that exosomes produced by ASCs contain a high concentration of cytokines like TGF-β and bFGF, which promote wound healing [33]. Moreover, Bour et al. and Davoodi et al. found that ASCs enhance fibroblast proliferation, new dermis formation, collagen production, and promote re-epithelialization in diabetic wounds [9,11].

Additionally, the determination of collagen deposition in the newly formed dermis aligned with the finding of the proliferative phase. We noted a significant increase in collagen density in the treatment groups, particularly in the groups treated with CTS, in comparison with control group. Nevertheless, one potential factor may be the composition of the CTS, which is predominantly made up of collagen [6]. However, studies have indicated that ASCs stimulate the production of cytokines, resulting in an increase in collagen density in the wound region [34,35]. So, we found that collagen density could be enhanced synergistically by the structure of CTS and the presence of exosomes derived from ASCs.

Angiogenesis and adequate blood supply are widely recognized as crucial elements that directly impact the process of wound healing [36]. Nonetheless, in diabetic ulcers, the blood flow diminishes considerably because of factors like vasculopathy, decrease in endothelial progenitor cells (EPCs), and impaired local cell function [21,37]. VEGF is the crucial cytokine in the process of angiogenesis. Localized cells, like fibroblasts, generate this factor, but in diabetes, high blood sugar and oxidative stress cause a notable decrease in their functionality [38]. Hence, we theorized that employing dermally sourced matrix-scaffold together with exosomes from ASCs, which are abundant in VEGF cytokine, could enhance angiogenesis in the wound area. To achieve this goal, the level of VEGF gene expression was measured, along with the number of blood vessels in the newly formed dermis, using stereological technique. In both aspects, we noticed a considerable rise in the treatment groups, particularly the CTS+Exo group than the control group. Bour et al. found that the dermal matrix includes VEGF cytokine and promotes blood vessel growth in the wound area [9]. Moreover, Shabbir et al. reported that exosomes obtained from mesenchymal stem cells have the ability to promote angiogenesis [39]. Notably, exosomes derived from ASCs contained a group of miRNAs linked to angiogenesis (miRNA-126, miRNA-130a, and miRNA-132) that aid in angiogenesis by increasing various growth factors like VEGF in endothelial cells [40,41]. Yu et al. found that exosomes derived from ASCs contain a high amount of VEGF mRNA [42]. Hence, we noticed that combining CTS and ASCs-derived exosomes simultaneously can have a synergistic effect on promoting angiogenesis at the wound location.

In addition to the molecular and cellular improvements observed, biomechanical analysis revealed that treatment groups, particularly the CTS+Exo group, exhibited significantly enhanced mechanical properties compared to the control group. The enhanced biomechanical properties of the CTS+Exo provide a crucial support for the tissue regeneration process. In diabetic wounds, where cellular function and tissue formation are compromised, maintaining scaffold stability is essential for proper tissue repair [22]. The increased maximum force and bending stiffness in the CTS+Exo group ensure that the scaffold can withstand the mechanical forces that arise during tissue remodeling and wound closure. A scaffold with sufficient mechanical strength promotes better cell migration, proliferation, and collagen deposition, which are essential for wound healing [5,43]. Furthermore, the improved stress high load and energy absorption in the CTS+Exo group suggest that this scaffold can better absorb and distribute mechanical stresses, thus enhancing the overall stability of the wound environment. These biomechanical improvements also correlate with the significant increase in collagen density and tissue strength observed in the treated groups. This is particularly important in diabetic wounds, where collagen synthesis is often impaired. By providing structural support to the newly formed tissue, the enhanced mechanical properties of the CTS+Exo aid in the proper alignment of collagen fibers, contributing to better wound closure and tissue regeneration.

Despite the promising results observed in our study, it is important to acknowledge several potential limitations and risks associated with CTS-exosome therapy. One potential concern is the possibility of immune responses triggered by the scaffolds. However, since the CTS used in this study was decellularized and almost completely devoid of DNA content, the likelihood of immune reactions is minimal. Decellularized scaffolds are generally considered to have reduced immunogenicity compared to those with cellular content, as they lack the major histocompatibility complex (MHC) markers that could trigger immune responses. Nonetheless, further studies are warranted to assess the long-term immunogenicity of CTSs.

Another challenge is the immune responses triggered by the exosomes, particularly if they are derived from allogeneic sources. Although efforts to minimize immune responses, such as careful isolation and purification of exosomes, have been made, further studies are needed to fully assess their immunogenicity and long-term effects on the immune system. Factors such as the method of isolation, storage conditions, and source of ASCs can influence the purity, quantity, and functionality of exosomes. To ensure reproducibility and reliability, standardized protocols for exosome isolation, and characterization should be established. Additionally, while the controlled biodegradability of the CTS is beneficial for sustained exosome release, the degradation byproducts could potentially have cytotoxic effects on the surrounding tissues. It is essential to evaluate the safety of these byproducts in long-term studies to ensure that they do not impede the healing process or cause adverse effects in vivo. In conclusion, while CTS-exosome therapy shows great potential for improving diabetic wound healing, it is important to address these potential risks and limitations in future studies to optimize the safety and effectiveness of the treatment.

## 5 Conclusion

In conclusion, the current research has effectively shown that the use of CTS together with ASCs-derived exosomes enhances the diabetic wound healing process. The biochemical composition and three-dimensional micro-porous structure of CTS are recommended for cell penetration, adhesion, migration, and angiogenesis. Furthermore, exosomes derived from ASCs act as crucial growth factors by preventing over inflammation and stimulating local cell differentiation, collagen synthesis, and angiogenesis.

## Ethics approval

All experimental protocols were approved by Ethics Committee of Mazandaran University of Medical Sciences, Sari, Iran (Ethic no: IR.MAZUMS.4.REC.1402.17171). All authors indicate that all animal experiments comply with the ARRIVE guidelines 2.0 (Animal Research: Reporting of in Vivo Experiments) and should be carried out in accordance with the U.K. Animals (Scientific Procedures) Act, 1986 and associated guidelines, EU Directive 2010/63/EU for animal experiments, or the National Institutes of Health guide for the care and use of Laboratory animals (NIH Publications No. 8023, revised 1978). Animals were kept in a laboratory standard hutch with no limitation access to a rodent's food and drinking water.

## Data availability

Data will be made available on request.

## Authors contributions

F.T.A. contributed to the study design, data acquisition and analysis, provided financial support, as well as drafting of the manuscript. M.O. contributed to isolation of dermal matrix and ASCs. S.S. designed molecular and tensiometrical assessments and analyses. A.D. stereological assessments. A. Kh. contributed to isolation and characterization of ASCs-derived exosomes. D.N. supervised the study and contributed to the study concept and design, interpretation of data, and editing and final approval of the manuscript. All authors reviewed and commented on the manuscript and approved the final manuscript.

## Funding

The current project was financially supported by grants to F.T.A. from Mazandaran University of Medical Sciences (Grant No: 17171), Sari, Iran.

## Declaration of competing interest

The authors declare no conflicts of interest.
